# Essential Oil of *Lavandula officinalis*: Chemical Composition and Antibacterial Activities

**DOI:** 10.3390/plants12071571

**Published:** 2023-04-06

**Authors:** Khaoula Diass, Mohammed Merzouki, Kaoutar Elfazazi, Hanane Azzouzi, Allal Challioui, Khalil Azzaoui, Belkheir Hammouti, Rachid Touzani, Flore Depeint, Alicia Ayerdi Gotor, Larbi Rhazi

**Affiliations:** 1 Laboratory of Applied and Environmental Chemistry (LCAE), Faculty of Sciences, University Mohammed Premier, Oujda 60000, Morocco; khaoula.diass@ump.ac.ma (K.D.); r.touzani@ump.ac.ma (R.T.); 2 Laboratoire de Chimie Appliquée et Environnement-Equipe Chimie Organique Macromoléculaire et Phytochimie, Faculté des Sciences, Université Mohammed Ier, Oujda 60000, Morocco; moh.merzouki@gmail.com (M.M.); allal.challioui@gmail.com (A.C.); 3 Agro-Food Technology and Quality Laboratory, Regional Center of Agricultural Research of Tadla, National Institute of Agricultural Research, Avenue Ennasr, BP 415 Rabat Principale, Rabat 10090, Morocco; kaoutar.elfazazi@inra.ma (K.E.); hananeazzouzi94@gmail.com (H.A.); 4 Laboratory of Engineering, Electrochemistry, Modeling and Environment, Faculty of Sciences, Sidi Mohamed Ben Abdellah University, BP 1796, Fez 30050, Morocco; k.azzaoui@yahoo.com; 5 Laboratory of Industrial Engineering, Energy and The Environment (LI3E) SUPMTI, Rabat 10000, Morocco; 6 Institut Polytechnique UniLaSalle, Université d’Artois, ULR 7519, UniLaSalle, 19 rue Pierre Waguet, BP 30313, 60026 Beauvais, France; flore.depeint@unilasalle.fr; 7 Institut Polytechnique UniLaSalle, AGHYLE, UP 2018.C101, UniLaSalle, 19 rue Pierre Waguet, BP 30313, 60026 Beauvais, France; alicia.ayerdi-gotor@unilasalle.fr

**Keywords:** *Lavandula officinalis*, essential oil, antibacterial activities, minimum inhibitory concentration (MIC), lavender, antimicrobial, molecular docking

## Abstract

The purpose of this study was to determine the chemical composition of the essential oil of *Lavandula officinalis* from Morocco using the GC-MS technique and assess the antibacterial effects against seven pathogenic bacteria strains isolated from the food origins of *Salmonella infantis*, *Salmonella kentucky*, *Salmonella newport,* three serotypes of *Escherichia coli* (O114H8K11, O127K88ac, O127H40K11) and *Klebsiella.* Tests of sensitivity were carried out on a solid surface using the Disc Diffusion Method. Results showed that *E. coli* and *S.newport* were sensitive to *Lavandula officinalis* essential oil. Minimum inhibitory concentrations (MIC) were determined using the method of agar dilution. The antibacterial results showed that four strains (three serotypes of *E. coli*, and *S. newport*) were remarkedly sensitive to *Lavandula officinalis* essential oil, giving MIC values of 88.7 µg/mL and 177.5 µg/mL. The molecular docking of the main oil products with the *E. coli* target protein 1VLY, showed that eucalyptol and linalyl acetate bind efficiently with the active site of the target protein. In particular, eucalyptol showed a higher activity than gentamicin used as positive control with a binding energy of −5.72 kcal/mol and −5.55 kcal/mol, respectively.

## 1. Introduction

Aromatic and medicinal plants are a source of several essential oils, and well-known since ancient times for numerous therapeutic properties [1,2]. *Lavandula officinalis* (Lavender) belongs to the Lamiaceae family specifically in the genus of Lavandula which consists of about 28 species [3,4]. Lavender is a shrub of height 20 to 80 cm that grows in sunny and mountainous areas of the Mediterranean, especially in the Rif, middle and high Atlas. It is spread over several regions in Morocco [5,6]. It is also cultivated in the southeast of the country [7]. The essential oil (EO) of *L. officinalis* have been used therapeutically for centuries for the treatment of several diseases, such as remedies for ulcers [8], scald [9], rheumatism [10], and nerve ache [10,11]. It is also recommended for cold [12], cough [13] and for diarrhea [14], as well as respiratory problems (e.g., asthma), fatigue, and convalescence [14,15,16]. It is used to moderate depression [17] as well as stress, anxiety, and insomnia [18,19]. It is known medicinally for its powerful antibacterial [20,21], anti-inflammatory, and analgesic properties, particularly for modulating pain and inflammation induced by formalin due to the inhibition of the COX enzymes [22]. It is a plant with various beneficial uses for the human body [9]. It is widely employed in the cosmetic industry in the production of skin care and cleansing lotions, scented bath soaps, perfume, and in the food industry to flavor drinks, ice cream, candy, baked goods and chewing gum [23].

Due to the propagation of the resistance phenomenon and the limited number of antibiotics under development, the discovery of new antibacterial agents has become more than essential [24]. There are many areas of research, but the exploration of natural resources appears to be the most promising because their biodiversity constitute, the largest reserve of active substances. The World Health Organization (2017) has established a global priority list of antibiotic resistant bacteria to facilitate prioritization of research and development of the new effective antibiotic treatments [25]. Several reports [21] have identified a variety of biological and/or pharmacological activities of some specific compounds of the *lavender* EO and observed that variations in the oil composition were, in general, associated with significant changes in activity, particularly antimicrobial activity [9,26,27,28].

This study deals with the identification of the chemical composition of *L. officinalis* EO, obtained from the aerial part of the plant originated and extracted in cooperative from Jerada (Morocco), and evaluation of the EO antimicrobial activity against seven pathogenic bacteria strains isolated from the food origins of *Salmonella infantis*, *Salmonella newport*, *Salmonella kentucky*, three serotypes of *Escherichia coli* and *Klebsiella*. These bacteria strains were chosen for their considerable pathogenic capacity and high contamination risk to food products.

This study may help to clarify whether lavender can be useful as an alternative or in combination to traditional antibiotic therapy.

## 2. Results and Discussion

### 2.1. Chemical Composition

**Figure 1:**
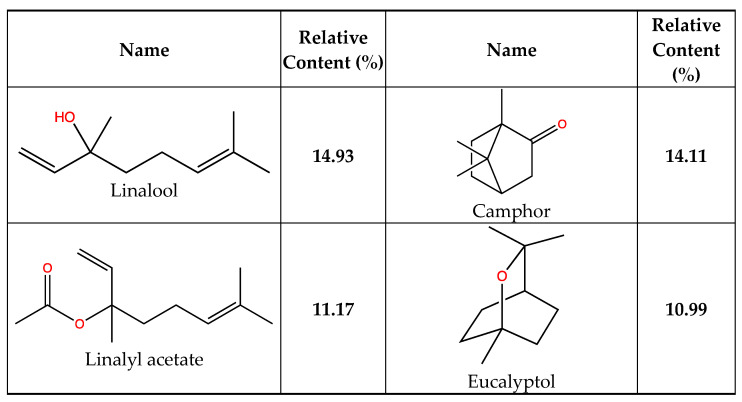
Developed formula and relative content of major *L. officinalis* essential oil identified compounds (Table 1).

The chemical composition of *L. officinalis* EO is presented in Table 1. A total of 31 compounds were separated using the GC-MS, representing 100% of the total EO. The main component was linalool (14.93%), and other significant compounds were camphor (14.11%), linalyl acetate (11.17%) and eucalyptol (10.99%) (Figure 1). Previous studies have shown that the qualitative and quantitative composition of the EO from the region of Rabat Salé-Zemour-Zaers had linalool (44.67%), linalool acetate (42%), 1.8-cineole (5.30%) and camphor (6.02%) [29,30] as main compounds while in another study from Meknes showed that the dominant compounds were linalool (29.95%), linalyl acetate (18.86%), ρ-cymene (14.68%), and alpha-campholenal (10.26%) [30]. In Azrou, located in the Middle Moroccan Atlas, the major constituents were linalyl acetate (44.96%) and linalool (44.64%) [31]. A previous study from Morocco showed different results, wherein the main components were composed of linalool (21.81%), 1,8-cineole (18.07%), camphor (11.89%), linalyl acetate (10.21%) [23]. These results already highlight that in Morocco, there exists a great genetic diversity of lavender which could be the origin of the composition variations in the essential oils. Besides the genetic diversity, the different climatic conditions in those three regions may also affect the composition. Thus, more investigations would be suitable for wide lavender screening in all regions of Morocco. Omurtag Özgen et al. [32], who examined the chemical composition of *Lavandula angustifolia* EO from Konya, Turkey, also yielded a different result as they found 28 compounds with a predominance of linalool (35.91%), 4-terpineol (6.10%), alpha-terpineol (4.49%) and lavandulol (2.49%) [28].

Another study from Turkey, working on the chemical identification and quantitative estimation of lavender EO, showed four main components: linalool (22.1%), lavandulyl acetate (15.3%), linalyl acetate (14.7%), and (E) beta-ocimene (10.4%), [33] whereas in this study lavandulyl acetate was not identified and beta-ocimene was a minor compound (Table 1). In the material assessed by Cong et al., 2009 [34], the EO of *Lavandula angustifolia* from Xinjiang, China, was characterized by a total of 17 compounds with linalool (44.54%), geraniol (11.02%), lavandulyl acetate (10.78%), 3,7-dimethyl-2,6-octadien-1-ol (10.35%), and isoterpineol (6.75%) as the main compounds, which is yet again a profile different to the Moroccan lavender analyzed. Data from the literature indicated that EO of *Lavandula angustifolia* from Wielkopolska (a region of Poland) contained linalool (24.6%) and (24.9%), linalyl acetate (14.4%) and (18.0%), borneol (6.2%) as the main compounds [35]. Two other studies in the same region were conducted. In the first study, the chemical composition of EOs from fresh and dried flowers and aerial parts of lavender were compared, where their main volatile components were revealed to be linalool (26.5–34.7%), linalyl acetate (19.7–23.4%), beta-ocimene (2.9–10.7%), and alpha-terpineol (2.8–5.1%) [36]. In the second study, 78 compounds have been identified where the main constituents were linalool (30.6%), linalyl acetate (14.2%), geraniol (5.3%), beta-caryophyllene (4.7%), and lavandulyl acetate (4.4%) [27]. Several works [37] have reported that the chemical composition of the EO is influenced by genotype, geographical origin [38], climatic conditions during growth [39], morphological characteristics, and propagation [40]. In addition, the quality of the lavender EO is based on its high content of linalool and linalyl acetate and their mutual proportions.

### 2.2. Antimicrobial Activity

The antimicrobial activity of the *L. officinalis* essential oil was tested using the disc diffusion method, and the results are presented in Table 2. The absence of microbial growth is reflected by a translucent halo around the disk, identical to sterile agar. In the literature on essential oils, the results of the aromatogram are expressed exclusively from the measurement of the diameter of inhibition halos in mm [41,42,43,44,45]. The sensitivity to oil was classified by the diameter of the inhibition halos (Table 2).

The antibacterial activity results revealed that the EO showed variable efficiency depending on the strain itself. The largest zone of inhibition with *L. officinalis* EO was obtained for all *E. coli* serotypes (O127H40K11, O127K88ac, O114H8K11) and *S. newport* serovar. However, *S. kentucky*, *S. infantis* and *Klebsiella* strains showed no sensitivity to *L. officinalis* EO for all the studied concentrations. Bacteria showing sensitivity to the EO were selected to determine the minimum inhibitory amount. The highest minimal inhibitory concentration (MIC) was obtained against *E. coli* (O127K88ac) and the lowest MIC was found for *S. newport* (MIC = 88.7 µg/mL) (Table 3). Interestingly, *L. officinalis* EO has also proved to be more active than gentamicin against all *E. coli* strains. In addition, the results of the microdilution test indicated that gentamicin is relatively more effective than *L. officinalis* EO against *S. newport* strains.

These significant differences in the sensitivity results demonstrate that the antibacterial activities depend on the bacteria species and strains. The results obtained are in agreement with those of other studies [46,47,48], as lavender essential oil has shown important antibacterial activities against different bacteria.

Investigation on the mechanisms of antibacterial action of the three monoterpenes showed that the antibacterial effect might partially result from a perturbation of the lipidic cytoplasmic membrane bilayer leading to the escape of the vital intracellular molecules [49,50,51]. The authors hypothesized that the antibacterial effect depended on the lipid composition and charge of the bacterial membrane surface. It seems that the lipopolysaccharide that covers the surface of the plasma membrane of the Gram-negative bacteria could explain the resistance of this type of bacteria to highly hydrophobic compounds by limiting their penetration into the membrane.

However, in our study the *E. coli* (*O127K88ac*) strain exhibited a very low MIC, thus demonstrating the presence of other mechanisms leading to the adsorption of the EO molecules onto the bacterial plasma membrane and therefore explaining the antibacterial effect. Thus, the defense mechanism of Gram-negative bacteria could partially be attributed to lipopolysaccharide molecules.

The antibacterial activities of the EO could be due to the antibacterial effect of one or various compounds. Additionally, a synergistic interaction between components may contribute to the effectiveness of EO against bacteria. Some studies have shown that whole EOs usually exhibit greater antibacterial activity than the mixtures of their major molecules, indicating that the minor components are crucial for the synergistic activity [50,51,52]. The antibacterial activities depend on the concentration of the EO compounds and its chemical composition. Recently [48], variability in the antibacterial activities of the EO from different cultivars of lavender was found, which corroborated with other investigations on different cultivars. Moreover, Predoi et al. [53] reported that the Gram-negative bacteria, *E. coli* ATCC 25922 and *E. coli* ESBL, were highly sensitive to the lavender EO, thus indicating a strong antimicrobial activity of lavender EO [52].

Correlation studies between chemical composition of lavender EO and antibacterial activities suggested the important antibacterial activities of linalool [48] confirmed by [54]. Linalyl acetate has also been found to have potent antibacterial properties [54]. Terpinen-4-ol, another compound present in our EO at about 8%, could be involved in the antibacterial effect because statistical analysis showed that this molecule is potentially responsible for antimicrobial activity [48]. Terpinen-4-ol has also been noticed to be efficient in destroying plasma membrane bilayer and increasing its permeability, leading to ion leakage and membrane dysfunction [55].

Camphor, one of the main compounds of our EO, could also contribute to antibacterial activities. Indeed, *L. pedanculata* and *L. dentata* essential oil, containing a high content of camphor (average of 51%) exhibited significant antimicrobial activity against different microorganisms [56]. It has also been determined that camphor is one of the most significant antibacterial bioactive compounds found in the *Achillea* species EO [57]. According to our results and these reports, higher antibacterial activities against the Gram-negative bacterial strains tested could be attributed partly to camphor molecule. Thus, bacterial defense mechanism based on the presence of lipopolysaccharides, is avoided by some EO molecules, such as camphor. Eucalyptol known as 1,8-Cineole, is the main compound of the studied EO. It is obviously involved in the establishment of the antibacterial activities of the EO. Various reports have shown that the essential oils with a high content of 1,8-cineole had moderate to strong antibacterial activity against numbers of microorganisms [58,59,60]. It was shown that, due to their synergistic effects, EO components could contribute to a significant variation in activity between them [29,59]. 1,8-Cineole tested in combination with other components (amoxicillin/clavulanic acid, and gentamicin) showed synergistic effects [61]. It was assumed that 1,8-cineole crosses the cell membrane and damages the cell organelles without structurally changing the membrane [55]. 1,8-cineole was found to block receptors that receive signals from several autoinducers and to have antibiotic activities [62]. It was shown that 1,8-cineole changes the shape and size of both Gram-negative and Gram-positive bacterial cells [63]. They showed that bacteria treated with 1,8-cineole caused a strong condensation of nuclear chromatin in the nucleoplasm, which led to cell apoptosis. The antimicrobial activity of eucalyptol is probably related to its hydrophobicity, making Gram-negatives susceptible to this compound. The action mechanism of this molecule is not yet clear. However, it was suggested that 1,8-cineole acts on an already disturbed bacterial membrane and somehow inactivates cellular components [54].

From these comparisons, we can conclude that the antibacterial activity of EOs depends not only on the content of active substances, but also on the chemical properties of these molecules, such as hydrophobicity and the bacteria itself.

### 2.3. Molecular Docking Studies

**Figure 2:**
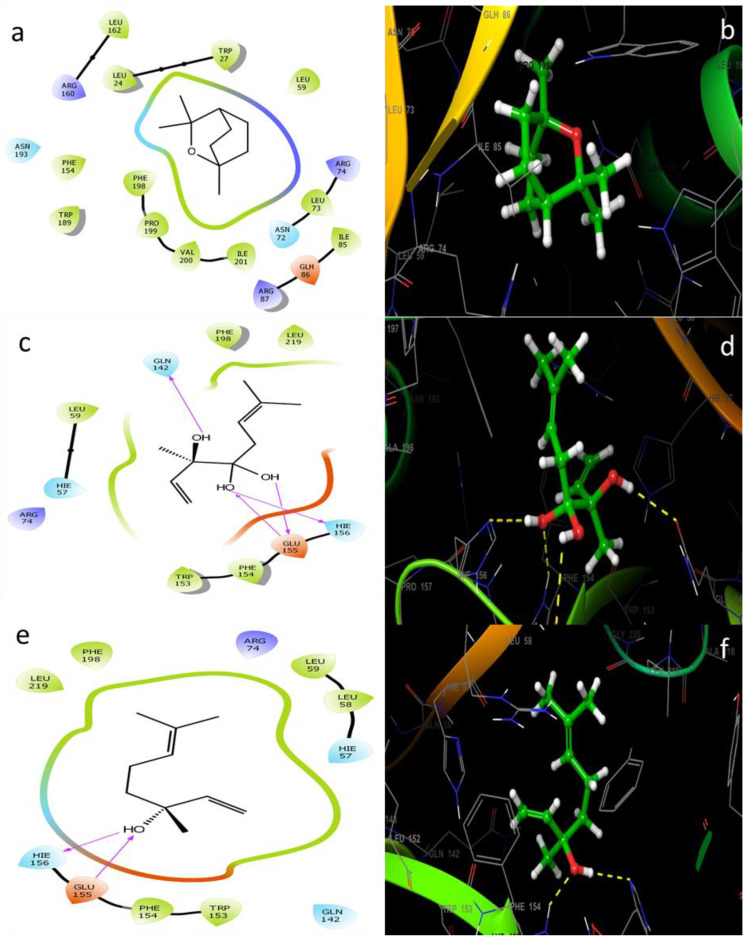
Intermolecular interactions 2D and 3D between (**a**,**b**) eucalyptol with *E. coli* 1VLY protein, (**c**,**d**) Linalyl acetate with *E. coli* 1VLY protein, (**e**,**f**) Linalool with *E. coli* 1VLY protein.

**Figure 3:**
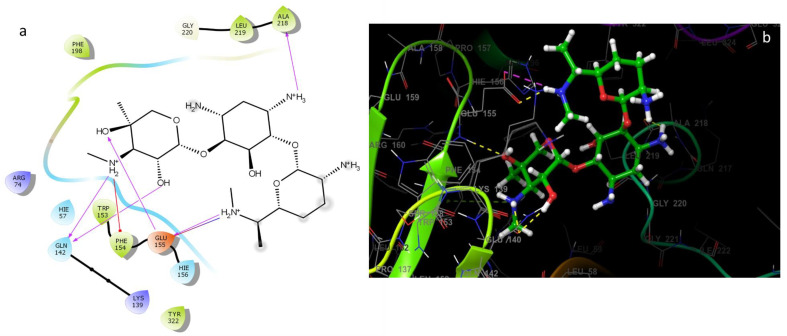
Intermolecular interactions 2D (**a**) and 3D (**b**) between gentamicin (control) with *E. coli* 1VLY protein.

The molecular docking approach can be used to model the interaction between a small molecule and a protein at the atomic level, which allows to characterize the behavior of small molecules in the binding site of target proteins as well as to elucidate fundamental biochemical processes [17,64]. The four compounds (linalool, linalyl acetate, camphor, and eucalyptol) (Figure 1) were selected for an in silico docking study with a bacterial protein from *E. coli*. All natural compounds have been effectively docked to proteins, with the exception of camphor. The glide score and hydrogen bond are estimated in Table 4. The two-dimensional ligand–protein images of the compound clearly show that all the compounds went inside the active binding site of protein cavity as projected in Figure 2 and Figure 3. The results of the docking simulation of the natural compounds (linalool, linalyl acetate, eucalyptol) and gentamicin (the positive control) in the active site of the 1VLY protein (Figure 2) showed that eucalyptol had a higher binding energy than the control and bound hydrophobic interactions with LEU24, TRP27, LEU59, LEU73, ILE201, VAL200, PRO199, PHE198, and ASN193 and other interaction with the positive-charged ARG74, respectively, with a docking score of −5.719 kcal/mol.

A higher energy binding score (−5278 kcal/mol) of the complex was found in the case of linalyl acetate which bound firmly to the active site of the protein and stabilized the active site through four conventional hydrogen bonds. Two conventional hydrogen bonds with HIS156, and two others with GLN142 and GLU155, were identified (Figure 2). Furthermore, the residues TRP153, PHE154, LEU219, PHE198, LEU59 were implicated in the hydrophobic properties.

Linalool bound to the active site through two conventional hydrogen bonds with a binding energy value of −3958 kcal/mol, higher than the control. The liaisons were HIE156 and GLU155, and the following residues LEU58, LEU59, PHE154 and TRP153, have been mainly implicated in the hydrophobic interaction.

The 1VLY protein belongs to the folate-dependent protein, YgfZ of *E. coli*. This family of protein participates in the biosynthesis and restoration of iron-sulfur (Fe-S) clusters. It uses folate to capture formaldehyde units and then help to preserve the activity of the Fe-S enzyme MiaB, a tRNA modification enzyme that is responsible of the methylthiolation of *N*^6^-isopentenyladenosine (i^6^A) to 2-methylthio-*N*^6^-isopentenyladenosine (ms^2^i^6^A) [65,66,67]. A previous report demonstrated that it is involved in the maintainence of the activities of the Fe-S enzymes succinate dehydrogenase, dimethyl sulfoxide reductase, 6-phosphogluconate dehydratase, and fumarase [67].

Results of molecular docking suggests that the EO components could inhibit the actions of all enzymes belonging to the folate-dependent protein family of YgfZ. The main compounds of our EO could also react with all proteins reacting with gentamicin. Thus, it is possible that some of these components could follow the same mechanism action of gentamicin that binds to the 16s rRNA at the 30 s ribosomal subunit, disturbing mRNA translation, and thus inducing the production of truncated or non-functional proteins [68].

The EO of *L. officinalis* could be used as a natural antimicrobial because it has various mechanisms of action, owing to the different bioactive molecules with different chemical properties.

## 3. Materials and Methods

### 3.1. Plant Materials

**Figure 4:**
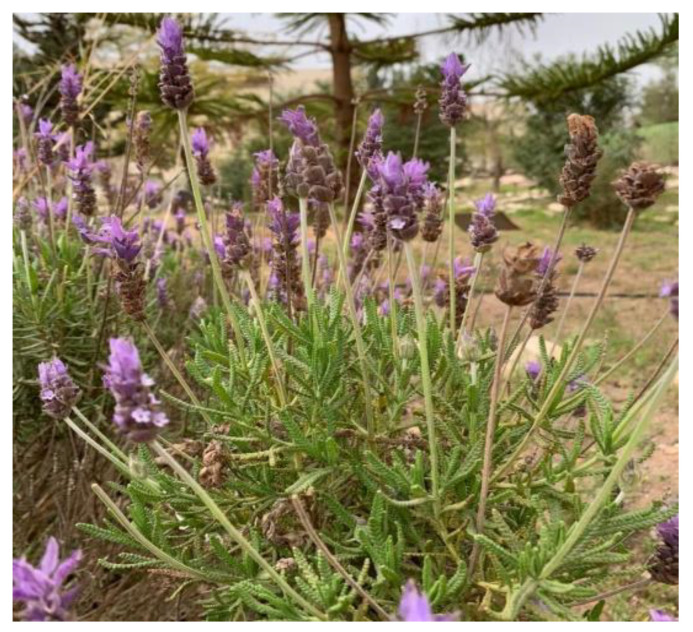
*L. officinalis*, (Lamiaceae) from Jerada, Morocco.

The aerial parts of the plant (leaves, stems, and flowers) of wild populations of *L. officinalis* (Figure 4) were manually collected in June 2018, in the Jerada Region (East of Morocco). The area of plant collection was characterized by short, hot and dry summers, and long, dry and cold winters with low precipitations, which is a typical Mediterranean climate.

### 3.2. Essential Oil Extraction

The EO was extracted in a local cooperative. The plant material was dried naturally on benches at room temperature (23–24 °C) for five days until the material was crispy. The EO was obtained after 2 h of steam distillation. The EO obtained was stored at 4 °C in tight vials until analysis.

### 3.3. GC-MS of Essential Oil of L. officinalis

**Figure 5:**
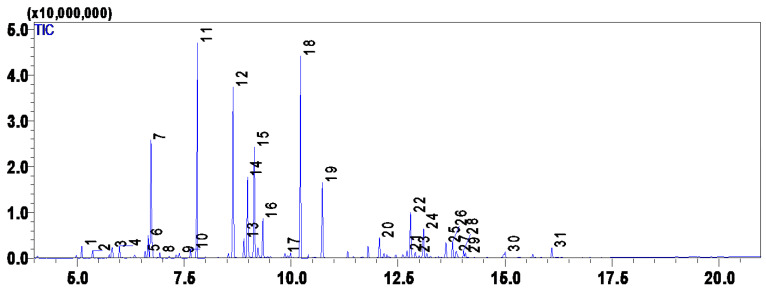
Gas Chromatography chromatogram of the essential oil of *L. officinalis* of Jerada (Morocco).

The obtained EO was analyzed using the gas chromatography with mass spectroscopy (Shimadzu GC-MS-QP2010, Japan), according to the protocol described previously by [10]. Helium inert gas was used as a carrier, and its pressure was adjusted to 100 KPa. Further modifications were realized, such as the oven temperature which was maintained at 50 °C for about 1 min, was subjected to a gradient of about 10 °C/min to reach an oven temperature of 150 °C. Afterwards, a gradient of 20 °C/min was set to reach a temperature of 250 °C. For the analysis of the samples, a quantity (1 µL) was taken from the EO and diluted in hexane. The injector was set at 240 °C on split mode with a ratio of (25:1). For the identification of the different constituents, a comparison of the retention time of each compound with its MS data using the National Institute of Standards and Technology (NIST) computer library was used [41,69,70] (Figure 5), following the same procedure as [71].

### 3.4. Antibacterial Assay

#### 3.4.1. Microbial Strain and Inoculum Preparation

Seven strains were used in the antimicrobial activity assessment of *L. officinalis* EO: three *Salmonella* serovars (*Salmonella newport*, *Salmonella infantis*, and *Salmonella kentucky*), three *Escherichia coli* serotypes (O114H8K11, O127K88ac, and O127H40K11) and *Klebsiella* strain. Bacteria were isolated from the food origins and identified at the Agro-food Technology and Quality Laboratory of INRA, Beni Mellal in collaboration with the Institute of Specialized Technicians in Agriculture (ITSA, Sidi Hammadi) located at the Béni Mellal region, Morocco. The above-mentioned bacterial strains were precultured in LB broth and incubated overnight at 37 °C. At 625 nm, the inoculum was adjusted at 0.5 Mc Farland, which corresponds to an optical density of 0.08 to 0.10. The inoculum had a final concentration of 10^8^ CFU/mL [72].

#### 3.4.2. Disc Diffusion Method

Antimicrobial activity of different essential oil extracted was evaluated using the disc diffusion method reported by [73,74]. The discs are made from Wattman paper, with a diameter of 6 mm. Then, these discs were put in a test tube, sterilized using the autoclave, and stored at room temperature. 20 mL of sterilized Muller Hinton agar (MHA) was poured into previously sterilized petri dishes and allowed to solidify. After solidification, 0.2 mL of the bacterial suspensions were spread on the surface under aseptic conditions.

The essential oils were freshly prepared and diluted in dimethylsulphoxide (DMSO). The discs were impregnated with 5 μL of the diluted sample (62, 5, 125, 250 and 500 μL/mL) of the essential oil. Paper disc moistened with DMSO solution has been used as reference control. The plates were then incubated at 37 °C for 24 h. After the incubation period, the zone of inhibition was measured and according to the method prescribed by Ponce et al. [74] the sensitivity to the EO was classified by the diameter of the inhibition zone: not sensitive (-) for diameters less than 8 mm; sensitive (+) for diameters from 8 to 14 mm; very sensitive (++) for diameters from 15 to 19 mm and extremely sensitive (+++) for diameters over 20 mm [71,74].

#### 3.4.3. MIC Determination

The minimum concentration (MIC) able to inhibit the growth of bacteria (absence of turbidity) was calculated using the microdilution broth method. Equal volumes of the diluted microbial culture were added to the EO, with concentrations ranging from 62.5 to 500 μL/mL. The optical density of the plate was measured at 625 nm. After incubating the plates at 37 °C for 24 h, the absorbance was measured at 625 nm. The MIC is the minimum value at which no growth is observed after incubation which means that the minimum value when the difference between the initial and final optical density is not significant [75]. Gentamicin was used as positive control. To investigate the significant difference between the results, several statistical analyses were performed after the normality test: ANOVA, and comparison of means. The groups were considered different when *p* < 0.05.

### 3.5. Ligand Preparation

Four natural compounds extracted from *L. officinalis* EO (Linalool, camphor, linalyl acetate, and eucalyptol) that can potentially be used as a drug were studied to detect their antibacterial activities against *E. coli*. gentamicin [75] was used as an antibacterial control obtained from the PubChem database (http://pubchem.ncbi.nlm.nih.gov accessed on 7 December 2022) [76], their 3D structure was prepared in an SDF format (structure-data file) and selected to perform molecular docking studies. Optimizations of the three-dimensional geometry with minimization of the energy of the ligand have been carried out using controlled algorithms in Maestro 12.8, Schrodinger 2021-2. The LigPrep module was applied by adding hydrogen atoms and eliminating salt and ionization at pH (7 ± 2), and the energy minimization was obtained using the OPLS_2005 force field [77].

### 3.6. Preparation of Protein and Molecular Docking

Docking studies have been carried out using the Schrödinger theoretical code to predict the biological behavior of compounds. Scoring functions and hydrogen bonds formed with the surrounding amino acids predicted the binding affinities. A negative and low value of the binding energy showed a strong favorable bond.

**Figure 6:**
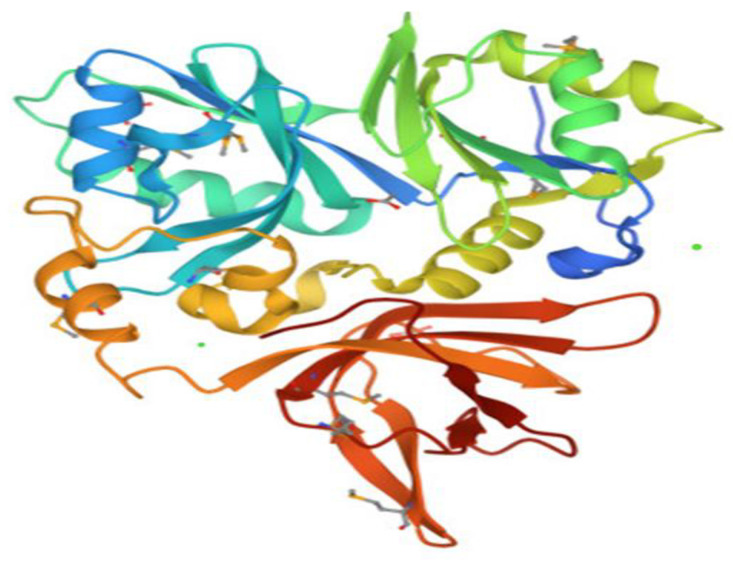
The crystal structure of *E. coli* (PDB ID: 1VLY).

The crystal structure of the *E. coli* with the crystalline structure (PDB ID: 1VLY) in Figure 6 had a resolution of 1.30 Å. The Rfree value was 0.168 while the Rwork value was around 0.134, which agreed well with the observed R-Value of 0.136. The proteins were selected and obtained from the PDB (Protein Data Bank) [78]. The protein structure was prepared using the Protein Preparation Wizard (Maestro 12.8, Schrodinger 2021-2, BioLuminate 2019). The ligand and water atoms were removed, as non-polar hydrogens were fused. While preparing the protein binding orders, personalized hydrogen atoms were also added and hot states using Epik: pH (7 ± 2) were generated. The minimization of the energy has been performed using the default RMSD limitation at 0.3 Å. The active site was chosen as the target center. The dimensions of the central grid box were chosen to include all atoms of the ligand set using the points (for x = 10, y = 10 and z = 10). Energy minimization was conducted by default, limiting the RMSD to 0.3 Å, and then the structure of the protein was minimized using the OPLS_2005 force field. Finally, the extra precision (XP) glide score was used to predict binding energy and to select anchored poses. Output docking scores were expressed as affinity binding (kcal/mol).

The best final conformation with the minimum binding energy was taken into account and converted into a two-dimensional and three-dimensional diagram showing the interaction of the ligand with the active site residue.

### 3.7. Statistical Analysis

All disc diffusion or the MIC assays were conducted with at least three repetitions per specie. Three to six measurements were performed for disc diffusion study on each bacterium to confirm results.

## 4. Conclusions

The chemical composition and the antibacterial activities of *L. officinalis* EO of the Jerada region (Morocco) was investigated in this study. After the extraction of the EO using the steam distillation method, the GC/MS analysis of the EO was carried out, and it was marked by dominance of four main components: linalool (14.93%), camphor (14.11%), linalool acetate (11.17%), and eucalyptol (10.99%). The antibacterial results showed that four strains (three serotypes of *E. coli*, *S. newport*) were remarkedly sensitive to lavender EO, giving MIC values of 88.7 µg/mL and 177.5 µg/mL. This EO may have many potential applications in food and pharmaceutical products. The in silico study of the antibacterial activity of the main products of the oil showed that eucalyptol and linalyl acetate are the effective products of the experimentally observed antibacterial activity against *E. coli,* which make these products a potential candidate for the treatment of *E. coli* infections. In particular, eucalyptol showed a higher antibacterial activity than gentamicin which was used as a positive control. However, further clinical and laboratory studies are required to confirm the therapeutic potential of these compounds.

## Figures and Tables

**Table 1:** The main compounds of the essential oil of *L. officinalis.* RT: Retention Time.

N°	Name	RT	% Area
1	alpha-Pinene	5.108	0.90
2	Camphene	5.362	0.63
3	Linalyl propionate	5.818	1.20
4	beta-Myrcene	5.989	0.90
5	beta-Cymene	6.589	0.57
6	D-Limonene	6.660	1.75
7	Eucalyptol	6.724	10.99
8	beta-cis-Ocimene	6.934	0.36
9	Linalool oxide	7.389	0.39
10	2-Carene	7.648	0.69
11	Linalool	7.814	14.93
12	Camphor	8.648	14.11
13	Lavandulol	8.898	1.59
14	Borneol	8.983	7.06
15	4-Terpinenol	9.140	8.31
16	p-menth-1-en-8-ol	9.341	3.29
17	Nerol	9.856	0.58
18	Linalyl acetate	10.223	11.17
19	Nerol acetate	10.731	5.55
20	Geraniol acetate	12.067	1.55
21	alpha-Santalene	12.710	0.68
22	Caryophyllene	12.794	3.71
23	alpha-Bergamotene	12.904	0.54
24	beta-Farnesene	13.096	2.49
25	Germacrene D	13.619	1.38
26	Linalyl propionate	13.776	1.27
27	cis-alpha-Bisabolene	13.860	0.91
28	gamma-Muurolene	14.032	0.61
29	beta-Sesquiphellandrene	14.076	0.42
30	Caryophyllene oxide	15.002	0.54
31	alpha-Bisabolol	16.094	0.93
Total identified			100

**Table 2:** Sensitivity of strains to lavender essential oil evaluated using the disc diffusion method. Classified as: not sensitive (-) for diameters smaller than 8 mm; sensitive (+) for diameters from 8 to 14 mm; very sensitive (++) for diameters from 15 to 19 mm and extremely sensitive (+++) for diameters over 20 mm.

Concentration (μL/mL)	*E. coli* (O127K88ac)	*S. newport*	*E.coli* (O114H8K11)	*E. coli* (O127H40K11)	*S. kentucky*	*S. infantis*	*Klebsiella*
**500**	+++	+++	+++	+++	-	+	-
**250**	++	+++	++	+++	-	-	-
**125**	+	++	+	++	-	-	-
**62.5**	-	+	+	+	-	-	-

**Table 3:** Minimal inhibitory concentration (MIC) in (µg/mL) of *L. officinalis* EO and gentamicin against bacteria. Each MIC value is the mean of three replicates ± SD.

	MIC in (µg/mL)	
Microbial strains	*L. officinalis* EO	Gentamicin
*Escherichia coli* (*O127K88ac*)	88.7 ± 0.02	200 ± 0.57
*Escherichia coli* (*O114H8K11*)	177.5 ± 1.13	200 ± 0.70
*Escherichia coli* (*O127H40K11*)	177.5 ± 0.82	200 ± 0.39
*Salmonella newport*	88.7 ± 0.9	63 ± 0.64
*Salmonella kentucky*	-	-
*Salmonella infantis*	-	-
*Klebsiella*	-	-

**Table 4:** The binding affinities and detailed interaction studies of lead compounds from *L. officinalis* with *Escherichia coli* 1VLY protein.

Compound	Binding Affinity (kcal/mol)	Interacting Amino Acid	Type of Intermolecular Bond
Linalyl acetate	−5.273	HIS156	Hydrogen bond
		GLN142	Hydrogen bond
		GLU155	Hydrogen bond
		TRP153	Hydrophobic
		PHE154	Hydrophobic
		LEU219	Hydrophobic
		PHE198	Hydrophobic
		LEU59	Hydrophobic
Eucalyptol	−5.719	LEU24	Hydrophobic
		TRP27	Hydrophobic
		LEU59	Hydrophobic
		LEU73	Hydrophobic
		ILE201	Hydrophobic
		VAL200	Hydrophobic
		PRO199	Hydrophobic
		PHE198	Hydrophobic
		ASN193	Hydrophobic
Linalol	−3.958	HIE156	Hydrogen bond
		GLU155	Hydrogen bond
		LEU58	Hydrophobic
		LEU59	Hydrophobic
		TRP153	Hydrophobic
		PHE154	Hydrophobic
Gentamicin	−5.552	GLN142	Hydrogen bond
		GLU155	Hydrogen bond
		ALA218	Hydrogen bond
		PHE154	Pi-cation
		GLU155	Salt bridge
		TRP153	Hydrophobic
		PHE198	Hydrophobic
		PHE154	Hydrophobic
		TYR322	Hydrophobic
		ALA218	Hydrophobic
		LEU219	Hydrophobic

## Data Availability

Not applicable.
